# Using a cognitive network model of moral and social beliefs to explain belief change

**DOI:** 10.1126/sciadv.abm0137

**Published:** 2022-08-19

**Authors:** Jonas Dalege, Tamara van der Does

**Affiliations:** Santa Fe Institute, 1399 Hyde Park Rd, Santa Fe, NM 87501, USA.

## Abstract

Skepticism toward childhood vaccines and genetically modified food has grown despite scientific evidence of their safety. Beliefs about scientific issues are difficult to change because they are entrenched within many interrelated moral concerns and beliefs about what others think. We propose a cognitive network model that estimates network ties between all interrelated beliefs to calculate the overall dissonance and interdependence. Using a probabilistic nationally representative longitudinal study, we test whether our model can be used to predict belief change and find support for our model’s predictions: High network dissonance predicts subsequent belief change, and people are driven toward lower network dissonance. We show the advantages of measuring dissonance using the belief network structure compared to traditional measures. This study is the first to combine a unifying predictive model with an experimental intervention and to shed light on the dynamics of dissonance reduction leading to belief change.

## Teaser

A cognitive network model tested in a longitudinal study shows that belief network dissonance predicts belief change.

## INTRODUCTION

The World Health Organization (WHO) lists vaccination hesitancy as one of the 10 greatest threats to global health (*1*). Erroneous beliefs about vaccines, which are somewhat common in the United States (*2*), can accelerate or even reignite the spread of diseases globally. In another recent report, the WHO also notes that around 45% of deaths among children less than 5 years of age are linked to undernutrition (*3*). Although the scientific community has shown that currently approved genetically modified (GM) crops are safe and could provide higher yields (*4*), many U.S. Americans are skeptical about this technology (*5*, *6*). Many other beliefs inconsistent with the scientific consensus, such as climate change denial, have similar detrimental consequences for society. Thus, it is critical for the scientific community to understand how these skeptical beliefs about scientific issues can be changed.

In this paper, we consider attitudes toward GM food and childhood vaccines as networks of connected beliefs (*7*–*9*). Inspired by statistical physics, we are able to precisely estimate the strength and direction of the belief network’s ties (i.e., connections), as well as the network’s overall interdependence and dissonance. We then use this cognitive network model to predict belief change. Using data from a longitudinal nationally representative study with an educational intervention, we test whether our measure of belief network dissonance can explain under which circumstances individuals are more likely to change their beliefs over time. We also explore how our cognitive model can shed light on the dynamic nature of dissonance reduction that leads to belief change. By combining a unifying predictive model with a longitudinal dataset, we expand upon the strengths of earlier investigations into science communication and belief change dynamics, as we describe in the next paragraphs.

Previous applied research on beliefs about GM food and childhood vaccines has found that skepticism about their safety is shaped both by relevant moral beliefs (e.g., care for others, concerns about the environment, and importance of naturalness and purity) and by perceived beliefs of trusted social groups, such as doctors or family members (*10*–*16*). Therefore, studies that focus on changing these beliefs have tried to vary the framing of the factual information and the source of the information, with mixed success (*17*–*20*). This literature sheds light on the importance of moral concerns and social groups in determining beliefs about GM food and childhood vaccines. However, these empirical studies tend to focus on specific interventions and populations (*21*) and do not draw on a unifying model to understand the processes that underlie belief change more generally.

Consistent with the findings of applied research on GM food and childhood vaccines, general models of belief change have identified two important sets of factors [for a review, see (*22*)]. First, people hold many personal beliefs, such as moral beliefs. In social psychology, the concept of dissonance was developed to understand when and why people might change their beliefs when they are incoherent with each other (*23*, *24*). Within this approach, incoherent beliefs lead to feelings of dissonance, and beliefs change if attention is then paid to these beliefs. Building on this concept of dissonance, more recent research has modeled the relationship between personal beliefs using cognitive network models to predict belief dynamics (*7*–*9*, *25*, *26*).

Second, people’s beliefs are shaped by their social networks. In statistical physics, models of opinion dynamics can predict change over time within a social network (*27*, *28*). However, these models generally do not take into account that the beliefs held by a person’s social network do not directly influence their own beliefs. Instead, their influence is mediated by how the person perceives beliefs in their social network (*29*–*32*). This implies that perceptions of beliefs in one’s social network can provide information that is different than the actual beliefs in one’s social network (*33*). For example, a person might overestimate how liberal their friends are and thus become more liberal themselves, just because their liberal friends voice their political position more firmly than their moderate friends.

In recent years, a few belief change models were developed to focus specifically on the interaction between personal beliefs (e.g., moral beliefs) and beliefs about one’s social network (social beliefs). These models tend to consider either the dissonance between moral and social beliefs using the concept of energy from statistical physics without considering the relationships between these beliefs (*34*), or the network imbalance between personal and social beliefs (e.g., belief A is positively connected to beliefs B and C, but beliefs B and C are negatively connected) (*35*–*37*). Dissonance-based models can explain belief change using estimated energies from reported moral and social beliefs (*38*). However, these models do not take into account the network structure of interrelated moral and social beliefs. On the other hand, models focusing on network imbalance can predict the final distributions of beliefs (*36*) and provide an explanation of how minorities can convince majorities (*35*), but empirical tests of whether these models can predict belief change are still lacking. Here, we do both: We suggest a unifying network model of interrelated beliefs and test it empirically with longitudinal data.

In this paper, we propose a network model that integrates moral and social beliefs to predict the dynamics of belief change. We extend and test the recent attitudinal entropy (AE) framework (*39*), a model inspired by statistical physics that conceptualizes individuals’ overall attitudes as networks of beliefs. For example, a person might have various beliefs related to their overall attitude toward childhood vaccines. They might be uncertain about their efficacy or concerned about unknown side effects, they might be distrustful of scientists who developed and studied these vaccines, or they might be wary of government health agencies that impose vaccine schedules. We consider all these moral and social beliefs to be part of a network and use characteristics of this network to predict belief change according to psychological theories of dissonance reduction.

The main parameters of our cognitive network model, their original representation in statistical physics, their corresponding psychological constructs, and the way we estimate them are listed in Table 1. As an analogy for the network of beliefs, we draw on a statistical physics model of an abstracted system of nodes, which are influenced by each other like magnetic spins.

**Table 1.** Overview of cognitive model parameters within the statistical physics framework and their corresponding psychological constructs and methods of estimation.

**Cognitive model parameter**	**Statistical physics term**	**Psychological construct**	**Estimation**
Network node state *b_i_*	Spin	Belief state	For each node *i*, we measure its value *b_i_* ∈ [ − 1,1]
Network tie ω*_ij_*	Coupling	Relationship between two beliefs	Partial correlation between *b_i_* and *b_j_* controlled for all other beliefs
Network dissonance *H*	Energy	Inconsistency between beliefs	Measures inconsistency (high dissonance) or consistency (low dissonance) of beliefs given estimated network structure. Sum of weighted distances of belief scores, *ω_ij_* ∣ *b_i_* − *b_j_*∣
Network interdependence β	Inverse of temperature	Any process that decreases randomness and disorder of belief networks such as attention and thought directed to the belief network	Average of the inverse of belief- specific scaling values of *b_i_* and *b_j_*, which are estimated in order to transform ω*_ij_* into measured correlations, with lower scaling values resulting in higher correlations. Higher interdependence reflects higher mean of the absolute correlations between beliefs

Social and moral beliefs related to the same issue are conceptualized as “nodes in the network,” which can take on different values. These represent spins (i.e., elements of the network that can take different states) in statistical physics. In the original AE framework (*39*), these spins are binary in nature. Here, we generalize to beliefs that can take any value between −1 and 1. Each possible value is a particular belief state that varies from complete disagreement to complete agreement with that belief.

“Network ties” between all moral and social beliefs, or “couplings” in the terminology of statistical physics, represent the strength and sign (positive or negative) of the relationship between beliefs. For example, with regard to beliefs about childhood vaccines, people who believe that vaccines are effective tend to trust scientists. Therefore, these two beliefs will have a strong and positive tie in the belief network. Within our cognitive model, each tie between two beliefs is estimated using partial correlations between these two beliefs controlling for all other beliefs in the network.

Given the nodes and their ties, we can estimate two sets of parameters at the network level. First, “the dissonance of the belief network” can be understood within the statistical physics framework as the energy of the system. Network dissonance refers to the actual inconsistency between all nodes in the network and is measured as the sum of the absolute difference between the values of a given node and a connected node, weighted by their tie. Here, we expand upon classic measures of dissonance (*23*, *24*), which only take into account the raw discrepancies between beliefs, by adding weights that represent estimated ties in the belief network.

Second, “network interdependence” reflects the statistical physics concept of temperature, with lower temperature corresponding to higher network interdependence. Network interdependence represents several important psychological processes that decrease randomness and disorder between beliefs, such as attention or thought directed to one’s beliefs (*39*). Network interdependence is estimated using the inverse of the average of scaling values that transform individual ties (estimated partial correlations) into measured correlations. Therefore, estimated interdependence is determined by the correlations between nodes, and higher correlations lead to higher estimated interdependence. When the network’s interdependence is high, there is little room for a node to be misaligned with its connected nodes.

**Fig. 1.**
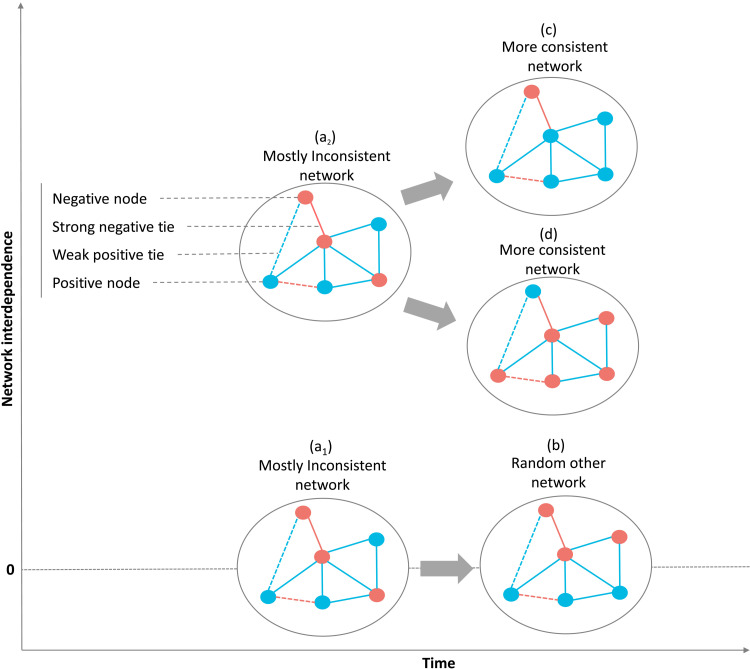
Cognitive network model of belief change. Network (a) is mostly inconsistent (high network dissonance) because it has some nodes that are in the same state but are connected by strong negative ties and some nodes that are in the opposite state but are connected by strong positive ties. When the network interdependence is zero (a_1_), the nodes will randomly change without achieving higher consistency (lower dissonance) (b). However, when the network interdependence is higher than zero, the mostly inconsistent (highly dissonant) network (a_2_) will change its nodes to achieve lower dissonance, either toward (c) or (d). The higher the network interdependence, the more likely the nodes in the belief network will change to achieve lower dissonance. Note that weak ties are less likely to lead to nodes changing because they have less of an effect on network dissonance.

The model assumes that given a minimum level of network interdependence, individuals aim to reduce their network dissonance by changing their beliefs (Fig. 1). Unless the network of beliefs is completely random (no interdependence), individuals will be driven to change their beliefs to align them with each other, thus lowering their overall network dissonance. This general tendency increases the more attention individuals pay to their beliefs, which causes network interdependence to increase. Within a statistical physics framework, systems are being driven to lower energy states, and the drive toward lower energy states is stronger at high inverse temperatures. This translates to psychological processes in the following way: When enough attention is directed to one’s beliefs, inconsistencies between beliefs will translate into greater felt dissonance, leading to belief change.

Returning to the example of beliefs about childhood vaccines, consider a person who believes vaccines to be ineffective but trusts scientists. These conflicting beliefs will contribute to network dissonance. However, this person might not be paying attention to the fact that these beliefs are conflicting and so nothing would happen. The more this person pays attention to their beliefs (network interdependence increases), the more likely it is that they will realize that these beliefs are not aligned (feel network dissonance), and they will be driven to change one of these beliefs. To reduce this dissonance, this person can change their beliefs about vaccines’ effectiveness (changing one node) or change their trust in scientists (changing another node). In principle, they could even change how they think scientists and effectiveness are related (that is, change the tie). However, in our current model, we do not focus on changes in the ties but only in changes in the values of the belief network nodes.

Changing beliefs is one of the central processes by which dissonance is reduced. This assumption is shared by both classic cognitive dissonance theory (*24*) and more modern consistency theories, such as constraint satisfaction theories (*8*, *40*) and the AE framework (*39*). We use the principles from these theories and present a network model of beliefs that enables us to estimate dissonance using the ties between belief nodes. We expect that using the structure of belief networks can increase our success in predicting belief change.

The relationships between different concepts in our cognitive network model can be expressed by Eqs. 1 to 3. First, for each node, we calculate its own network dissonance

$$H_{i}=\sum_{j;j\neq i}\omega_{\mathrm{ij}}|b_{i}-b_{j}|$$ (1)

where *H_i_* is the network dissonance of node *i* in the belief network (moral or social), *b_i_* is the value of the node, and ω*_ij_* is the tie between *b_i_* and another node in the network, *b_j_*. The belief network dissonance *H_i_* is the sum of the absolute distances between *b_i_* and all other nodes weighted by the ties. In this paper, we focus on the ties, thus omitting the external field from the network dissonance equation usually found in statistical physics models. Note that this network dissonance equation differs from the network dissonance equation used in the original AE framework. We use Eq. 1 to account for continuous beliefs.

Weighting network dissonance by the ties has the advantage that the resulting network dissonance implicitly takes the structure of the belief network into account. For example, a highly inconsistent but strongly connected belief network would have higher network dissonance than a highly inconsistent but only weakly connected belief network. Similarly, a belief represented by a node that is central (i.e., a node with many strong ties) in the network but inconsistent with other nodes would result in higher network dissonance than a belief represented by a peripheral node (i.e., a node with mostly weak ties) that is inconsistent with other nodes.

The conditional probability that a given node will change is

$$P(b_{i}\to b_{i}^{\prime })=\frac{1}{1+e^{\beta \Delta H_{i}}}$$ (2)

where Δ*H_i_* = *H_i_*′ − *H_i_* is the change in network dissonance between the two node values (*H_i_*′ = −∑_*j* : *j* ≠ *i*_*ω_ij_b_i_*′*b_j_*), and β is the network interdependence. The probability of the node changing value from *b_i_* to *b_i_*′ increases with (i) the difference in network dissonance between the new value and the current value when the new value is lower (Δ*H_i_*), and (ii) the increase in interdependence of the entire network (β). The higher the network interdependence, the higher the probability of belief change toward a new state with lower network dissonance.

In addition to calculating the probability of a given belief changing, we can also calculate the probability that we find the whole network in a given configuration of belief states

$$P(b)=\frac{e^{-\beta H(b)}}{Z}$$ (3)

where *P*(*b*) is the probability of finding a given configuration of node values, *H*(*b*) is the sum of all node-specific dissonances [*H*(*b*) = ∑*_i_H_i_*], and *Z* is a normalizing constant ensuring probabilities add up to 1.

In sum, belief change is predicted to be more likely when individuals have high network dissonance given some network interdependence. In other words, individuals will try to achieve greater consistency between their beliefs to reduce feelings of dissonance created by inconsistencies between highly interconnected beliefs if individuals pay attention to these inconsistencies. To test the role of network dissonance for belief change, we need longitudinal data on beliefs over time, from which we can estimate an empirical model of belief networks drawn from our cognitive network model. Investigating the dynamics of dissonance reduction goes beyond the usual investigations of cognitive dissonance, which usually only analyzes the consequences of inducing dissonance (*41*–*43*). Here, we investigate how dissonance interacts with receiving new information on a topic and whether such new information leads to reconfiguration of one’s beliefs leading to lower dissonance. We also test whether taking into account the network structure of beliefs improves the predictability of belief change compared to classic measures of dissonance.

## RESULTS

We used a nationally representative longitudinal study of beliefs about GM food and childhood vaccines to test whether the belief network dissonance can help shed light on belief change processes (see Materials and Methods for details on study design and questionnaire). This study included questions about both moral beliefs related to each technology [e.g., GM food (childhood vaccines) are beneficial to children, GM food (childhood vaccines) are part of our tradition] and social beliefs about their safety [e.g., percentage of medical doctors believe that GM food (childhood vaccines) is (are) safe, and percentage of my family and close friends believe that GM food (childhood vaccines) is (are) safe; see table S1 for all questions). We assessed these beliefs four times over three waves of data collection (over an average of 30 days): once in the first and third waves and twice in the second wave (before and after an intervention). In the second wave, we presented individuals with an educational intervention on the safety of GM food and vaccines, quoting reports from the National Academies of Sciences. Participants were divided into five experimental groups for the GM food study and four experimental groups for the study of childhood vaccines. We had one control condition in each study where participants did not receive any intervention. All experimental conditions received the same scientific message about safety with a different framework (for the full list of educational interventions, see table S2). A total of 979 individuals participated in all three waves and answered all relevant questions for the study.

### Estimation of the cognitive network

To fit our cognitive network model to empirical data, we focused on variations in all moral and social beliefs after removing variations explained by individual-level and time-level differences (see Materials and Methods for details). We also made three key assumptions. First, we assumed that Gaussian distributions are appropriate for our data (see empirical support in fig. S1), which allowed us to estimate a Gaussian graphical model (GGM). Second, we assumed that our data represented an equilibrium distribution. Although individual beliefs can change, we used a fixed distribution of all beliefs in the belief network. Third, we assumed that ties in the estimated group-level belief networks were representative of the ties at the individual level (see empirical support in figs. S2 and S3).

Our model can be specified in several different ways when fitted to empirical data. We did not make any assumptions about (i) whether the ties, intercepts, and/or network interdependence vary over time and (ii) whether the networks are sparsely or densely connected. To investigate whether our constructs vary over time and whether network interdependence increases during the time course of the study, we fitted different specifications of our model on the four time points to see if we needed to constrain partial correlations representing ties in the network to be equal across time points, constrain intercepts to be equal across time points, and/or constrain network interdependence to be equal across time points (see Materials and Methods for details on network estimation). In addition, we tested whether the data can be captured best by a dense network (all beliefs are directly connected to all other beliefs) or a sparse network (some beliefs are not directly connected). The results indicated that a sparse network with equal partial correlations and equal intercepts between time points and varying network interdependence between time points fits the data best for both attitudes toward GM food and childhood vaccines (see table S3 for fit measures of each specification). This implies that the network structure remained constant throughout time, but the interdependence between beliefs varied.

**Fig. 2.**
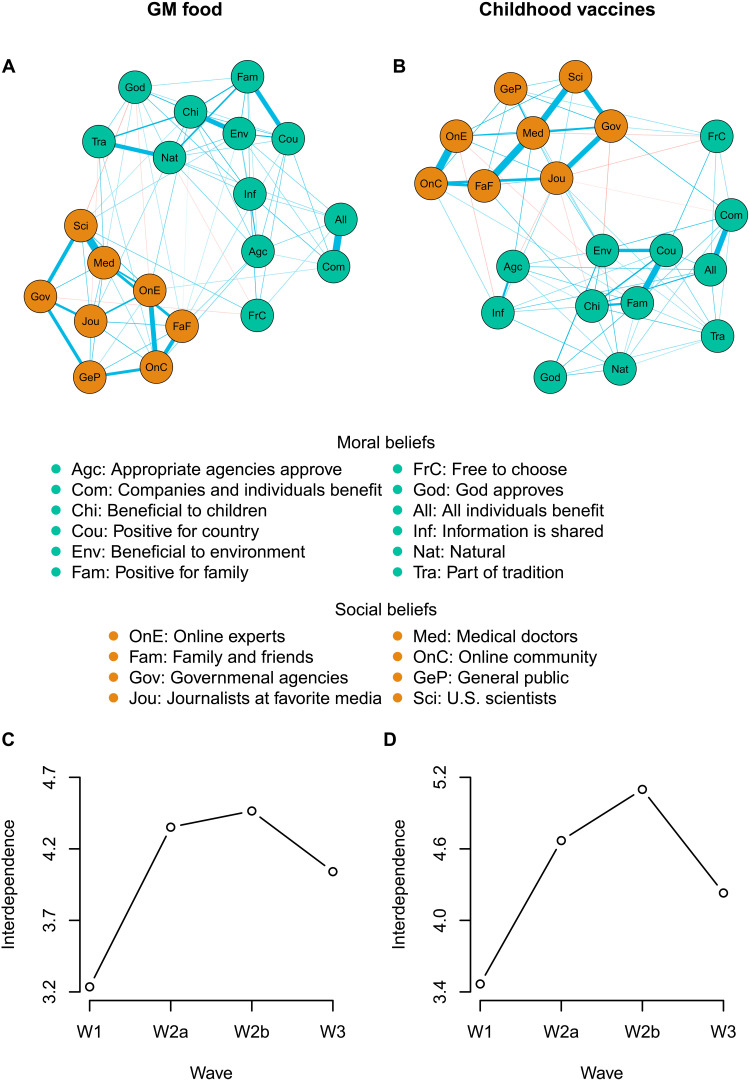
Belief networks and development of interdependence over measurements. The networks are shown for GM food (**A**) and childhood vaccines (**B**) and include moral beliefs (orange nodes) and social beliefs (green nodes). The ties represent the partial correlations between two beliefs controlled for all other beliefs. Blue (red) ties represent positive (negative) correlations, and the widths of the ties correspond to the strength of the correlations. The strength of the ties ranged from 0.02 (between the beliefs “Chi” and “Fam”) to 0.30 (between the beliefs “Med” and “Sci”) for GM food and from 0.02 (between the beliefs “Com” and “Jou”) to 0.28 (between the beliefs “OnE” and “OnC”), *N* = 979. (**C**) and (**D**) show the development of network interdependence over measurements for GM food and childhood vaccines, respectively.

Estimated group-level networks for beliefs about GM food and childhood vaccines are shown in Fig. 2 (A and B). In both networks, moral and social beliefs were connected to each other but formed two distinct clusters. Most beliefs were positively connected, but there were also some negative connections. For example, there was a negative estimated tie between the belief that scientists think GM food is safe and the belief that God approves of GM food. The network representing attitudes toward GM food was more densely connected than the one representing attitudes toward childhood vaccines, indicating that beliefs toward childhood vaccines were more independent of each other. This could indicate that people have more nuanced beliefs about childhood vaccines than about GM food.

According to our model, the relationship between network dissonance and belief change holds only if network interdependence is greater than zero. To check that this was the case here, we estimated the networks’ interdependence, a measure of attention or thought toward beliefs. We found that the belief network interdependence was higher than zero and increased sharply between wave 1 and wave 2 [see Fig. 2 (C and D)]. As participants were drawn to pay attention to their beliefs, their beliefs became more interconnected. Therefore, we expect that participants were driven to change their beliefs to reduce their overall belief network dissonance. After wave 2, network interdependence remained relatively constant and decreased slightly between wave 2 and wave 3. This development might reflect a ceiling effect reached in wave 2, after which participants again paid less attention to their beliefs.

### Beliefs about GM food and childhood vaccines over time

**Fig. 3.**
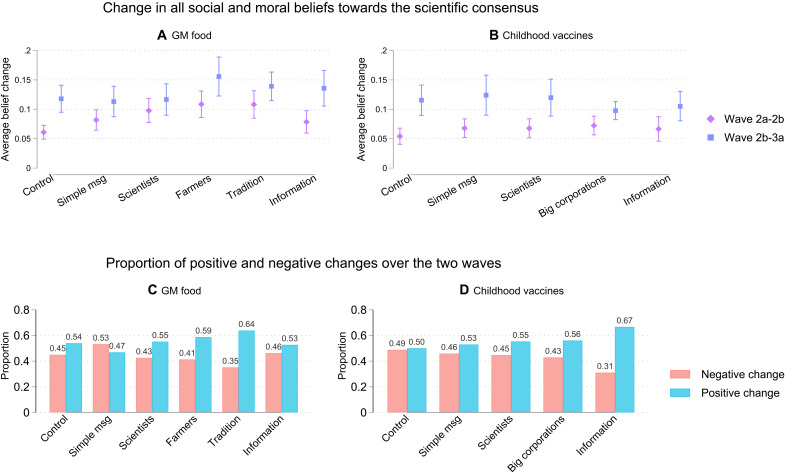
Network beliefs over time. Average raw beliefs over time for groups receiving different educational interventions about GM food (**A**) and childhood vaccines (**B**) and proportion of participants who showed on average a positive and negative change in beliefs regarding GM food (**C**) and childhood vaccines (**D**). *N* = 979.

The main test of our cognitive network model is whether network dissonance can predict belief change. Before conducting this test, we provide a descriptive overview of changes in beliefs during the course of our study. Figure 3 shows the average change in beliefs between wave 2a and wave 2b (before and after the experiment) and again between wave 2b and wave 3 (on average, 10 days apart). We calculated the average of all social and moral beliefs (higher values represent more positive views toward GM food or childhood vaccines) and then took the difference between measurements. We focused on changes in averages of beliefs because we were interested in changes in the overall state of the belief network rather than changes in beliefs that cancel each other out (e.g., one belief becoming more positive, while another belief becomes more negative). In other words, changes in the averages of beliefs reflect changes in the general attitude of someone toward the subject at hand. In addition to the change in the overall state of the belief network, we also investigated whether belief-specific dissonances can predict changes in individual beliefs. We focused on belief change within wave 2 and between wave 2 and wave 3, because the participants received the intervention during wave 2.

On average, all participants were more accepting of the scientific consensus over time, but the effects of educational interventions were minimal. Before and after the experimental intervention (Fig. 3, A and B, purple dots), even people in the control group moved toward greater acceptance of GM food and childhood vaccines. Ten days later (Fig. 3, A and B, blue dots), there was an even greater increase in the acceptance of both GM food and childhood vaccines. There were, however, little differences between the groups. For participants who were interviewed about the safety of GM food, participants who received a framing focused on scientists, farmers, and tradition had a greater increase in their acceptance of GM food before and after the experiment compared to participants in the control group (*t* − test : *p*(*m*_sci_ − *m*_cont_ < 0.001), *p*(*m*_farm_ − *m*_cont_ < 0.001, *p*(*m*_trad_ − *m*_cont_ < 0.001))). Educational interventions focusing on farmers and tradition were also better than receiving the simple message about the safety of GM food (*t* − test : *p*(*m*_farm_ − *m*_cont_ < 0.05, *p*(*m*_trad_ − *m*_cont_ < 0.05))). There were no significant differences between the groups in wave 2b and wave 3. For vaccines, there were no significant differences between the groups during the experiment (wave 2a and wave 2b) or 10 days later (wave 2b and wave 3). The larger change in beliefs between wave 2b and wave 3 compared to between wave 2a and wave 2b for all groups could reflect that participants’ beliefs became less interdependent between wave 2 and wave 3 (see Fig. 2, C and D). As attention toward one’s belief decreased, participants were more likely to change beliefs randomly. The rather minimal overall effect of the educational intervention could be due to a mix of success and backlash among participants after receiving new information on GM food and vaccines.

Although, on average, people changed their beliefs toward more positive views of GM food or vaccines, quite a few participants moved toward more negative beliefs. To note, only 15 participants did not change any of their beliefs over time. In our sample, 61.72% on average had changes in their beliefs toward more accepting (positive) beliefs about GM food and childhood vaccines. However, 38.28% of our participants changed their beliefs on average toward more skepticism (negative beliefs). This type of backlash is quite common in studies of beliefs about GM food and childhood vaccines (*19*, *44*). Beliefs about GM food were more likely to change negatively compared to beliefs about childhood vaccines. However, according to our cognitive network model (Fig. 1), the relationship between network dissonance and belief change should hold regardless of the direction of belief change.

Given the overall mixed success of educational interventions, we turn to our cognitive network model, which can be used to explain belief change over time and in either direction. In the next sections, we test whether belief network dissonance is associated with belief change and the conditions under which participants accept or reject the information from the scientific intervention.

### Network dissonance predicting belief change

To test whether belief network dissonances predicted belief change, we first calculated each individual’s network dissonance and belief-specific network dissonance. We multiplied the estimated network tie between any two given beliefs by the absolute difference between the recorded responses each individual had on these beliefs. The belief network dissonance is then the sum of these pairwise network dissonance scores, and the belief-specific dissonance is the sum of all pairwise dissonances of the given belief (see Materials and Methods for more details on their calculations). Second, we calculated correlations between estimated network dissonance and belief change separately for GM food and childhood vaccines for each experimental intervention group to account for potential differences in responses to the framing. We tested whether dissonances predict the average change in all beliefs and whether belief-specific dissonances predicted the change in specific beliefs. In addition, we tested whether dissonances predicted belief change within wave 2 after the intervention and whether dissonances predicted belief change between wave 2 and wave 3.

### Network dissonance predicting average belief change

We first calculated correlations between network dissonance and belief change for each scientific issue and within each intervention group and then combined these correlations in a meta-analysis.

#### During the educational interventions

**Fig. 4.**
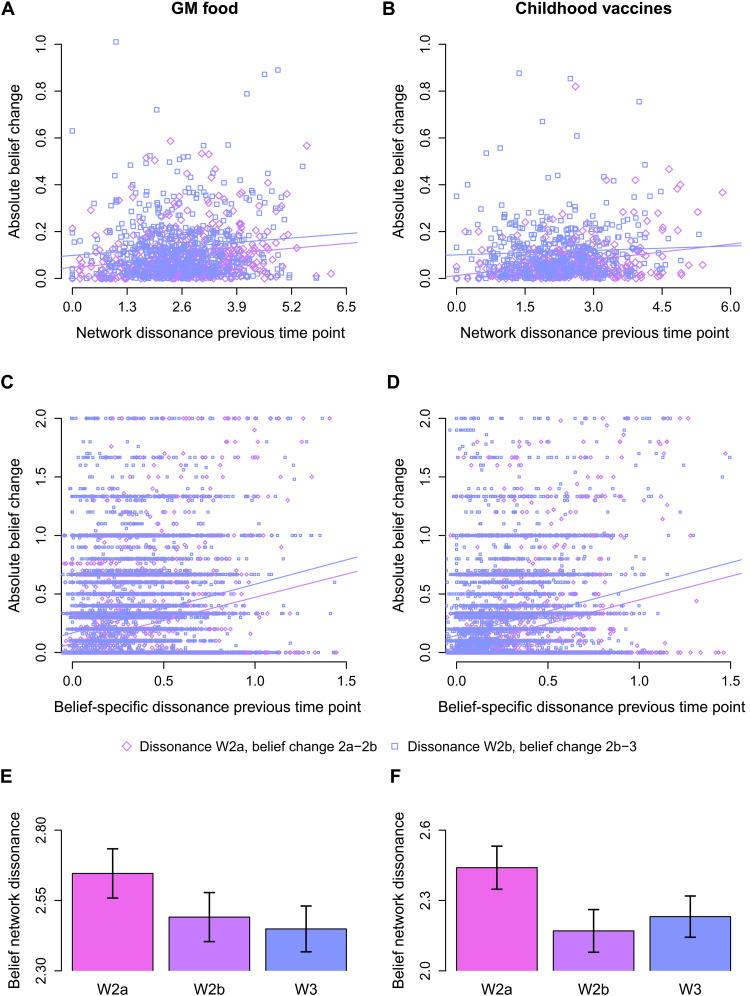
Network dissonance and belief change before and after the intervention. (**A**) and (**B**) show the relationship between network dissonance and absolute belief change for GM food and childhood vaccines, respectively, where each dot represents a participant. (**C**) and (**D**) show the relationship between belief-specific dissonance and absolute belief-specific change for GM food and childhood vaccines, respectively, where each dot represents a single belief of a participant. (**E**) and (**F**) show the belief network dissonance before and after the interventions in wave 2 and the follow-up measurement in wave 3. Error bars in (E) and (F) indicate 95% confidence intervals, *N* = 979.

The meta-analysis of the correlations between network dissonance (estimated before the intervention) and belief change during the intervention showed a weak to moderate positive correlation for both GM food (see Fig. 4A) and childhood vaccines (see Fig. 4B), *r* = 0.24, *P* < 0.001 (see table S4 for correlations per experimental group). The meta-analysis also showed that there was significant heterogeneity in the correlations between the different groups, *Q*(10) = 36.16, *P* < 0.001. We therefore proceeded to test whether correlations differed between beliefs about GM food and childhood vaccines and whether the control conditions differed from the experimental conditions. These moderators did not affect the magnitude of the correlation between belief network dissonance and belief change, *Q*(2) = 0.86, *P* = 0.650. Individuals with a higher belief network dissonance were more likely to change their beliefs compared to individuals with a lower belief network dissonance. This was the case for both beliefs toward GM food and childhood vaccines, and belief change was equally well predicted when individuals received an intervention or not.

#### Between wave 2 and wave 3

The meta-analysis of the correlations between network dissonance (estimated at the end of wave 2) and belief change between wave 2 and wave 3 showed a weak positive correlation for both GM food (see Fig. 4A) and childhood vaccines (see Fig. 4B), *r* = 0.13, *P* = 0.001 (see table S4 for correlations per experimental group). The meta-analysis also showed that there was no significant heterogeneity in the correlations between the different groups, *Q*(10) = 8.75, *P* = 0.556. In general, network dissonances predicted belief change between wave 2 and wave 3, but somewhat less accurately compared to within wave 2. This weaker effect might be due to the rather long time interval between the waves and how individuals might have changed their beliefs in a more random fashion.

Together, belief network dissonance predicted belief change on a short time scale (within a study wave) but to a lesser extent on a longer time scale (between study waves). Our model suggests that the reason for the unpredictability of belief change on longer time scales might be due to low belief network interdependence, which leads beliefs to change in a somewhat random fashion.

### Belief-specific dissonance predicting belief-specific change

In addition to testing whether we can predict belief change at an average level using network dissonance, we also tested whether we can predict the change in specific beliefs. We calculated multilevel correlations, taking into account that each individual has 20 different correlations between dissonances and beliefs. We did this separately for each topic and each intervention and then again combined the different interventions in a meta-analysis.

#### During the educational interventions

The meta-analysis of the correlations between belief-specific dissonance (estimated before the intervention) and belief change during the intervention showed a weak to moderate positive correlation for both GM food (see Fig. 4C) and childhood vaccines (see Fig. 4D), *r* = 0.23, *P* < 0.001 (see table S5 for correlations per experimental group). The meta-analysis also showed that there was significant heterogeneity in the correlations between the different groups, *Q*(10) = 36.16, *P* < 0.001. We therefore proceeded to test whether the correlations differed between beliefs about GM food and childhood vaccines and whether the control conditions differed from the experimental conditions. These moderators did not affect the magnitude of the correlation between belief-specific dissonance and belief change, *Q*(2) = 1.04, *P* = 0.59. Individuals with higher belief-specific dissonance were more likely to change their beliefs compared to individuals with low belief-specific dissonance. This was the case for both beliefs toward GM food and childhood vaccines, and belief change was equally well predicted when individuals received an intervention or not.

#### Between wave 2 and wave 3

The meta-analysis of the correlations between belief-specific dissonance (estimated at the end of wave 2) and belief-specific change between wave 2 and wave 3 showed a weak to moderate positive correlation for both GM food (see Fig. 4C) and childhood vaccines (see Fig. 4D), *r* = 0.19, *P* < 0.001 (see table S5 for correlations per experimental group). The meta-analysis also showed that there was significant heterogeneity in the correlations between the different groups, *Q*(10) = 34.63, *P* < 0.001. Therefore, we proceeded to test whether the correlations differed between beliefs about GM food and childhood vaccines and whether the control conditions differed from the experimental conditions. The omnibus test of these moderators was significant, *Q*(2) = 9.23, *P* = 0.010. Although the type of scientific issue did not moderate the magnitude of the correlation between dissonance and belief change, *b* = − 0.02, *P* = 0.26, whether the individuals were in an experimental condition did moderate the correlation, *b* = − 0.07, *P* = 0.006. We therefore conducted two separate follow-up meta-analyses. Belief-specific dissonances predicted belief change better in the control conditions, *r* = 0.25, *p* < .001, than in the experimental conditions *r* = 0.18, *p* < .001. Individuals with higher belief-specific dissonance were more likely to change their beliefs compared to individuals with low belief-specific dissonance, and this effect was more pronounced for individuals who did not receive an intervention.

Together, belief-specific dissonance predicted change in specific beliefs on a short time scale as well as on a longer time scale (between study waves). This finding contrasts with the finding that average belief change was not as well predicted on a longer time scale. We found a similar pattern of results for the prediction of belief change between wave 1 and wave 2a (see fig. S5). The reason for the more robust prediction of belief-specific change might be that these changes are less affected by variations in network interdependence than average belief change.

### Reduction of belief network dissonance

To investigate whether people changed their beliefs to reduce their belief network dissonances, we tested whether individuals’ belief network dissonances decreased over time. Given that belief network interdependence was highest during wave 2 and decreased between wave 2 and wave 3, we expected that the decrease in belief network dissonances is more pronounced within wave 2 than between wave 2 and wave 3.

#### Before and after the educational interventions

We first tested whether belief network dissonances decreased during the interventions. A meta-analysis that combined beliefs toward GM food and vaccines and all groups showed that network dissonances were lower at the end of wave 2, and the effect was moderate, *d* = 0.31, *P* < 0.001 (see table S6 for differences per experimental group). There was significant heterogeneity in the effect sizes, *Q*(10) = 20.50, *p* = .025. Therefore, we included the type of scientific issue and whether the participants were in the control group or in an experimental group as moderators. The omnibus test of the moderators was not significant, *Q*(2) = 5.77, *P* = 0.056, but the type of scientific issue was a significant moderator, *b* = 0.19, *P* = 0.017. Whether individuals were in the control group or in an experimental group was not a significant moderator, *b* = 0.02, *P* = 0.836. Therefore, we conducted two separate meta-analyses for GM food and childhood vaccines. We found a significant weak positive effect for GM food (see Fig. 4C), *d* = 0.22, *P* < 0.001, and a significant moderate positive effect for childhood vaccines (see Fig. 4D), *d* = 0.43, *P* < 0.001. Individuals reduced their dissonances more for childhood vaccines than for GM food.

#### Between wave 2 and wave 3

We first tested whether belief network dissonances decreased during the interventions. A meta-analysis combining beliefs toward GM food and childhood vaccines and all groups showed that network dissonance did not reliably decrease or increase from wave 2 to wave 3, *d* = 0.00, *P* = 0.901 (see table S6 for differences per experimental group). The effect sizes were not significantly heterogeneous, *Q*(4) = 8.59, *P* = 0.572. Belief network dissonances fluctuated only randomly between wave 2 and wave 3.

Together, these analyses indicate that when individuals focused their attention on their beliefs (their belief network interdependence increased; see Fig. 2), they reduced their dissonances (Fig. 4, E and F). This occurred not only during the intervention but also between the beginning of wave 1 and wave 2a (fig. S5). It seems that participants paid more attention to their beliefs right after being first prompted to think about them and also after seeing new information. However, whether because of lack of questioning or because of a decrease in interest in the study, participants had a decrease in interdependence (Fig. 2) and subsequently an increase in dissonance (Fig. 4, E and F) after the end of wave 2.

All in all, we found that network dissonance, belief change, and interdependence relate to each other over time, in line with our model assumptions. Interventions aimed at changing people’s beliefs led to a reconfiguration of beliefs that allowed people to move to lower network dissonance states and more consistent belief networks. However, these reconfigurations were not always in line with the objective of the intervention and sometimes even reflected a backlash.

### Predicting positive versus negative belief change

**Fig. 5.**
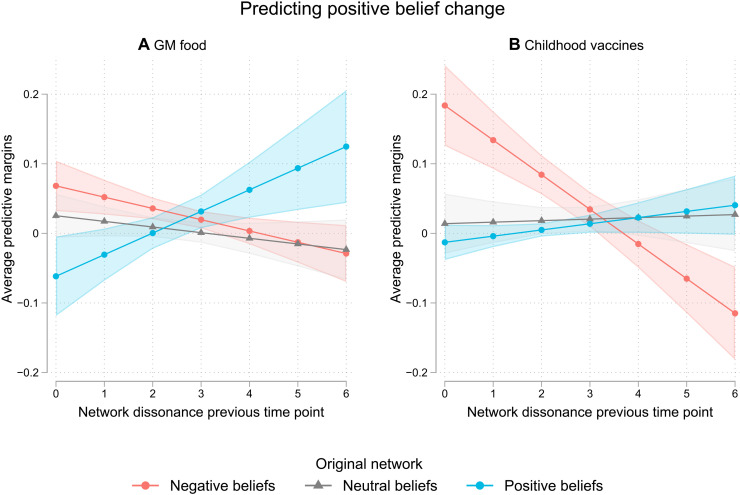
Belief change toward the scientific consensus by network dissonance and original beliefs. For participants who received the educational intervention on the safety of GM food (**A**) and childhood vaccines (**B**), results from mixed-effect models for waves within each individual, with random intercept for each individual and wave, controlling for the framing and demographics. The areas represent 95% confidence intervals, *N* = 979.

Although our measure of network dissonance, and especially belief-specific dissonance, predicts absolute belief change, belief change did not always lead to greater acceptance of the scientific consensus for participants receiving educational interventions. We proceeded to explore whether other factors might influence the direction of belief change and found that initial beliefs interact with dissonance to predict the change in direction of beliefs. Figure 5 shows the average predictive margins of positive belief change for people with originally a more negative belief network (average less than 3.5 on the scale of 1 to 7, *N*_GM_ = 252 and *N*_Vac_ = 43), an overall neutral belief network (average between 3.5 and 4.5, *N*_GM_ = 207 and *N*_Vac_ = 87), or a positive belief network (average over 4.5, *N*_GM_ = 90 and *N*_Vac_ = 300). We tried different cutoffs and a continuous measure and found similar results. We estimated these predictive margins using a multilevel linear model with the two waves nested within each individual, controlling for experimental group, gender, family status (children or not), and political values to also test the robustness of our findings. The average predictive margins were estimated using the predicted value of belief change averaged across all people in our sample. The resulting average marginal effect (AME) is the difference between the average predictive margins of belief change across network dissonance values.

For GM food, the effect of the network dissonance estimated in the previous wave on belief change was strongest for people who had originally positive beliefs [AME_diss ∣ pos_ = 0.186; 95 % confidence interval (CI),0.055 to 0.318] and was significantly greater than the effect of dissonance for people with originally negative beliefs (AME_diss ∣ pos/neg_ = 0.284, *P* < 0.001) or neutral beliefs (AME_diss ∣ pos/neut_ = 0.235, *P* < 0.01). Participants who were initially positive about GM food and had a high network dissonance changed their beliefs toward even greater acceptance. Neutral individuals had fewer changes in their beliefs, and their belief change was not predicted by network dissonance (AME_diss ∣ neut_ = − 0.049; 95 % CI, − 0.118 to 0.021). Among participants who were more negative toward GM food, network dissonance was associated with a stronger change toward more negative beliefs (AME_diss ∣ neg_ = − 0.097; 85 % CI, − 0.170 to − 0.024).

The general pattern holds for participants who received an educational intervention targeting the safety of childhood vaccines, but the details vary. Although there was a positive trend between dissonance and belief change for participants with positive beliefs, this effect was not significantly different from zero (AME_diss ∣ pos_ = 0.053; 95 % CI, − 0.012 to 0.118). This was the same for participants initially neutral about childhood vaccines (AME_diss ∣ neut_ = 0.013; 95 % CI, − 0.076 to 0.102). However, participants with originally negative beliefs had a greater belief change toward negative beliefs when their network dissonance was high compared to when their network dissonance was low (AME_diss ∣ neg_ = − 0.299; 85 % CI, − 0.413 to − 0.184).

Together, these findings indicate that individuals reduced their belief network dissonances in a way that was more influenced by their initial beliefs than by the content of the interventions. Generally, participants changed toward more extreme beliefs when prompted to think about their beliefs rather than changing their beliefs according to the information they received during the intervention.

### Model comparison

A central feature of our cognitive network model is that the dissonance calculation takes into account the structure of the belief network. This is the case for both the overall network dissonance and belief-specific dissonances. A crucial question that arises is whether taking into account the network structure of beliefs into account improves predictability of belief change. To compare our model to models that do not take into account the structure of the network of beliefs, such as classical dissonance models and recent formal models of dissonance (*22*–*24*), we tested whether the correlations between dissonances and belief change decrease when dissonances are not weighted by the network ties. To this end, we calculated unweighted dissonances between beliefs, with all ties set to 1. We then tested whether the correlation between these unweighted dissonances and belief change was lower than the correlation between weighted dissonances and belief change. The results of these analyzes are shown in Table 2. As can be seen, weighted dissonances generally outperformed unweighted dissonances. Only for the prediction of the average belief change between wave 2b and wave 3, the weighted and unweighted dissonances did not differ significantly from each other. In all other comparisons, weighted dissonances predicted belief change significantly better than unweighted dissonances. Taking the network structure of beliefs into account does improve the predictability of change in beliefs.

**Table 2.** Comparison between weighted and unweighted dissonances for predicting belief change.

**Predicted belief change**	**Weighted dissonances**	**Unweighted dissonances**
Average change between wave 2a and wave 2b	*r* = 0.22	*r* = 0.16∗
Average change between wave 2b and wave 3	*r* = 0.09	*r* = 0.07
Belief-specific change between wave 2a and wave 2b	*r* = 0.27	*r* = 0.20∗
Belief-specific change between wave 2b and wave 3	*r* = 0.23	*r* = 0.21†

*Correlations differ significantly at the *P* < 0.001 level.

†Correlations differ significantly at the *P* < 0.01 level.

## DISCUSSION

In this paper, we show that dissonance estimated from a cognitive network model can predict belief change. Expanding on the AE framework (*39*), we propose a cognitive network model that combines social and moral beliefs. This model enables us to precisely estimate important predictors of belief change, such as the relationship between beliefs (ties), their overall dissonance (network dissonance), and the attention toward beliefs (network interdependence). We used a nationally representative longitudinal survey to estimate network models for beliefs about GM food and childhood vaccines. We find that the dissonance estimated from the network model can be used to predict both average changes in the entire belief networks and changes in specific beliefs. Furthermore, we explore how the direction of belief change is partially explained by people’s original attitudes toward GM food and childhood vaccines. Last, we show that weighting dissonances by estimated network ties improved the predictability of belief change compared to more traditional measures of dissonance.

Conceptualizing the relationship between beliefs as a network can help us understand the mechanisms that lead to belief change. Using our cognitive network model, we investigated the dynamics of network interdependence (i.e., how much influence beliefs exert on each other) and the dynamics of network dissonance reduction. Network interdependence increased immediately after participants were prompted to think about their beliefs, with only slight changes during and after the second wave of the study, and even a decrease in attention at the very end of the study. Network dissonance reflected these changes in attention over time. Participants decreased their network dissonance during right after the first wave and during the interventions, underscoring our model’s implication that attention and thought lead to reductions in dissonance. Although our cognitive network model is only an analogy for actual cognitive processes, these findings show its usefulness in estimating and disentangling key psychological factors that influence belief change.

We have three main contributions. First, we combine social and moral beliefs into a single cognitive network model built through a statistical physics framework. This model extends our recent proposed framework for unifying moral and social beliefs (*22*) by also taking into account the network structure of all beliefs. This model draws on previous research that combines moral and social beliefs (*34*) and uses network models for the relationships between beliefs (*35*, *36*). Previous research on belief formation and change has stressed the importance of both sets of factors as individuals make decisions. However, due in part to the lack of cross-disciplinary research, the combination of both sets in one framework remains rare. In this paper, we draw on social psychology and statistical physics to not only incorporate beliefs across these two domains but also include them as part of an interacting network. We hope that this research encourages more studies of the interactions between social and moral belief networks as important determinants of belief change.

Second, our cognitive network model is able to empirically predict belief change by connecting physical parameters to actual psychological constructs. Many belief dynamic models have remained untested with empirical data. In addition to a formal model, we provide empirical predictions about belief change using data collected specifically to answer these questions. We ground our model in social psychology, thus bridging the gap between belief dynamics models in statistical physics and empirical work on science communication. We develop clear psychological meanings for statistical physics parameters and test their empirical validity. Belief network dissonance provides a formalization of dissonance, and network interdependence provides a formalization of attention directed at an issue. This enables us to illuminate some of the mechanisms behind belief change. Individuals are motivated to reduce the dissonance between beliefs and reconfigure their beliefs to allow for lower dissonance. Such a reconfiguration can be, but is not necessarily, in line with the aim of the intervention. The direction in which individuals change their beliefs depends not only on the intervention but also on the easiest way for individuals to reduce their dissonance. This finding also goes beyond the classic finding that inducing dissonance leads to belief change (*41*–*43*) by showing that providing individuals with new information interacts with dissonances in their belief network. Individuals with low dissonance are unlikely to change at all, whereas individuals with high dissonance can change in both directions.

Third, we contribute to the study of dissonance and belief change by showing that the predictability of belief change improves when one takes into account the network structure of beliefs. This insight is an important step toward a more fine-grained understanding of when beliefs are likely to change and gives more credence to modeling beliefs as networks. We emphasize that representing beliefs as networks is not only a descriptive tool but also can shed light on why some beliefs are easier to change than others. An interesting avenue for future research would be to estimate a belief network for each person, so that we get an even more fine-grained understanding of the dynamics leading to belief change.

There are some limitations to this study. First, we estimated network interdependence per time point for the entire group of participants because current network estimation methods cannot estimate network interdependence separately for each individual. The group-level network interdependence thus likely represents the average network interdependence of the group with individual variation possibly captured by variations in network dissonance. A longer longitudinal study and more advanced methods would enable individual-level estimates of network interdependence. Second, we did not have an empirical measure of attention, and so we could only infer that our estimated measure of network interdependence was related to attention through other proxies. However, network interdependence could reflect many other psychological processes that lead belief networks to move to more consistent states or not. Third, as discussed above, our model predicted absolute belief change, but not the direction of belief change, toward either acceptance or rejection of the safety of GM food and vaccines. Future research should expand on this model to provide ways to explain why some individuals accept or reject an experimental intervention and if individuals are choosing the “easiest” path to a more consistent belief network. We provided a first step in this direction, with our exploratory analysis on predicting whether individuals change toward more positive or negative beliefs, but more research is necessary to attune to the direction of belief change. Last, we focused on cognitive beliefs of one individual at a time; however, individuals are connected within larger social networks that influence the dynamics of belief change over a large population. We hope that subsequent research will continue to bridge social psychology and statistical physics to model and test belief change at the individual and societal levels.

We encourage future research to further investigate the details of our model in addition to the general mechanisms specified in the current paper. These investigations could focus on individual variation in belief network structures. For example, it is likely that people who attach high importance to their beliefs have more densely connected belief networks (*25*). Further investigations into such individual variation are likely to enable us to further specify the general principles of our cognitive belief network model. Another promising investigation for future research would be to further investigate how belief network dissonance interacts with interventions targeting specific nodes. One could, for example, first estimate a person’s belief network dissonance and then administer an intervention specifically targeted at a belief that is high in dissonance. We expect that this would result in a stronger belief change than administering an intervention aimed at a belief with low estimated network dissonance.

This research has implications for science communication on issues critical to the health of many. Science communication should take into account that directing attention to a given topic leads to higher network interdependence, which, in turn, leads to higher needs for dissonance reduction. This reduction of dissonance can work against the objective of the intervention if individuals have beliefs that are incongruent with the intervention. We expect that educational scientific interventions that focus on reducing the dissonance of the belief network will be more effective in changing the minds of science skeptics. Designing these interventions is not an easy task, but targeting interventions specifically to the structure of belief networks might prove effective. For example, beliefs that already have high network dissonance will be easier to change than beliefs that have lower network dissonance. Further research to translate our findings into science communication might help combat erroneous and socially harmful beliefs.

## MATERIALS AND METHODS

### Experimental design

We conducted a longitudinal study with an experimental component over three waves within a probabilistic national sample in the United States. The study was exempted from institutional review board (IRB) oversight by the University of New Mexico (IRB no. 16018). To select participants for the study, we screened *N* = 2482 participants for their beliefs about the safety of GM food and childhood vaccines. We selected *N* = 1832 participants who were somewhat hesitant about the safety of GM food or vaccine for the main experimental study. In other words, we only included individuals who selected a number between 1 and 6 (included) for the screener question, “Do you think it is unsafe or safe to eat GM food?” or “Do you think childhood vaccines are unsafe or safe for healthy children?” with the options from 1 (completely unsafe) to 7 (completely safe).

Of the 1832 selected participants, 979 completed the three waves with no missing values on any relevant questions. We only included these participants who had no missing values in our analyses (ages 23 to ≥90; median, 60; female/male, 568/411). The first wave, on average 90 days after the screener, questioned participants about their beliefs about the safety of GM food and childhood vaccines, as well as related moral concerns and perceived beliefs of social contacts and sources. These questions were then administered again in wave 2, on average 20 days later, both before and after an experimental manipulation, and again in wave 3, on average 10 days later.

To measure individuals’ moral and social beliefs about GM food and childhood vaccines, we included questions about related moral beliefs (*45*) and the perception of participants of the beliefs of relevant social groups (*46*). Haidt and Kesebir (*47*) identify six moral foundations relevant for different groups of U.S. Americans: care, fairness, loyalty, authority, purity, and liberty. We developed two questions for each of the moral foundations. For the social network, we focused on perceived beliefs about the safety of GM food or vaccines from direct social contacts (family, close friends, and online community) and relevant sources of information (medical doctors, scientists, governmental agencies, online influencers, journalists, and the U.S. general public). The full list of questions about related moral and social beliefs is in the table S1.

We included other questions in each wave of the questionnaire for external validation of our model. We developed three questions that focused on felt dissonance. These questions asked if the participant felt at ease, unbothered, and comfortable (all also on a scale from 1 to 7 and recoded so that higher values indicate higher dissonance). We averaged these three items into an index of felt dissonance. Cronbach’s alphas in the different waves were high for both GM food and childhood vaccines (GM food wave 1: 0.93, wave 2: 0.93, and wave 3: 0.94; childhood vaccines wave 1: 0.92, wave 2: 0.93, and wave 3: 0.95), indicating high reliability.

In the second wave of the survey, participants were randomly assigned to different experimental groups that received scientific facts about GM food and vaccines combined with messages that addressed different social and moral considerations. Supplementary materials include the experimental conditions for participants selected for the GM food study (*N* = 549) and the childhood vaccines study (*N* = 430) (see table S2). Each message in the GM and vaccines surveys had similar levels of readability and word count.

### Network estimation and calculation of network dissonance

We estimated belief networks including moral and social beliefs for GM food and childhood vaccines separately. Before estimating the networks, we regressed each belief on each person and time points to partial out these effects. In other words, we wanted to predict belief change unexplained by individual-level or time-level differences. We then used the residuals of these regression analyses to estimate the networks.

We implemented our theoretical model using the GGM, which is the most common approach to estimate networks from continuous data. Edges in a network represent partial correlations between two nodes while controlling for all other nodes. The modeling of the variance-covariance matrix Σ can be done in the following way (*48*)

$$\Sigma =\Delta {\left(I-\Omega \right)}^{-1}\Delta$$ (4)

where Ω represents the partial correlations between the nodes and measures the ties ω of our cognitive network model. Δ represents a diagonal scaling matrix with square roots of the diagonal precision matrix scaling the partial correlations on the diagonal and 0 s on the off-diagonal. These scaling values measure the inverse of the network interdependence $\frac{1}{\beta }$ of our model. The difference between these scaling values and inverse network interdependence is that there is one scaling value for each belief, while there is a single value for inverse network interdependence in our model. The reason to have a separate scaling value for each belief is that scaling a GGM by a single value often results in a variance-covariance matrix that is not positive definite. Lower scaling values result in higher correlations between beliefs, because the model-implied correlations result from dividing the partial correlation between two given beliefs by the product of their scaling values. To have a measure of network interdependence, we took the inverse of the mean of these scaling values.

We fitted the networks separately for GM food and childhood vaccines across the different time points and increasingly constrained the parameters of the specifications of our model in eight steps. We then assessed the fit of these eight different specifications based on the Bayesian information criterion. These specifications were estimated using the R package psychonetrics (*49*). We first let all parameters vary freely between time points and then constrained the following parameters to be equal across time points: partial correlations between nodes (Ω), intercepts of the nodes, and scaling values (Δ, as a proxy of network interdependence). We included constraints in the intercepts because this allowed us to use an approach similar to testing measurement invariance and made variations in the scaling values identifiable. We tested each constraint using either a dense network (all nodes connected) or a sparse network (some ties set to 0). We determined which ties were set to 0 using a prune step-up procedure, which sets a given tie to 0 and tests whether this results in a better or worse model fit. We then selected the best-fitting specification of the model.

For both the GM food and childhood vaccine networks, the best fitting specification of our model was a sparse model (i.e., some partial correlations between beliefs were set to 0) with equal partial correlations across time points (i.e., partial correlations between all beliefs were set to the exact same values at every time point) and intercepts but unconstrained network interdependence across time points (see table S3 for fit measures of the different specifications of the model), implying that the network structure remained constant over time, while network interdependence varied over time.

To calculate belief network dissonance per person at each time point, we used estimated partial correlations of the belief network. We multiplied the partial correlation between any two given beliefs by the absolute difference between the recorded responses each individual had on these beliefs. The belief network dissonance is then the sum of these pairwise network dissonance scores, and the belief-specific dissonance is the sum of all pairwise dissonances of the given belief.

### Test of assumptions and validation

To test the appropriateness of our assumption that Gaussian distributions fit our data, we investigated whether the multivariate distributions of the measured beliefs confirmed to a normal distribution. As can be seen in the fig. S1, this assumption was met.

To test the suitability of our assumption that the estimated ties at the group level were representative of the ties at the individual level, we first investigated the relationship between the individual and the group variances. The results of this analysis are shown in fig. S2. We found that questions with high individual-level variance over time tended to also have higher group variance, measured at one time point. Second, we compared correlations between beliefs estimated over all time points without taking into account the multilevel nature of our data with correlations controlling for the multilevel nature of our data (with time points nested within individuals). As can be seen in the fig. S3, only the absolute size of the correlation coefficients was affected by the different estimation methods. Correlation coefficients were considerably higher when the multilevel nature of the data was not taken into account (GM food: mean *r* = 0.39; childhood vaccines: mean *r* = 0.41) compared to when it was taken into account (GM food: mean *r* = 0.24; childhood vaccines: mean *r* = 0.28). However, the relative size of the correlations between beliefs was remarkably similar between the different forms of estimations, indicating that the group-level correlations were similar to the individual-level correlations. This was also confirmed by almost perfect correlations between the different estimation methods (GM food: *r* = 0.98, *P* < 0.001; childhood vaccines: *r* = 0.97, *P* < 0.001). Taking these findings together, we concluded that the ties estimated at the group level were representative of the ties at the individual level.

In another test of the validity of our model, we checked whether estimated belief network dissonances correlated with self-reports of felt dissonance. We found generally weak correlations between estimated belief network distances and self-reports of felt dissonance, *r*_GMfood,W1_ = 0.10, *P* = 0.022, *r*_GMfood,W2_ = 0.06, *P* = 0.173, *r*_GMfood,W3_ = 0.06, *P* = 0.189, *r*_vaccines,W1_ = 0.11, *P* = 0.035, *r*_vaccines,W2_ = 0.18, *P* < 0.001, *r*_vaccines,W3_ = 0.20, *P* < 0.001. However, these weak correlations are likely due to our inadequate measure of felt dissonance. Upon reexamining this measure, it appeared that the questions were not formulated in a sufficiently specific way. This resulted in questions that measured general affect toward the topic of the study and not actual feelings due to contradicting beliefs. This was supported by the finding that the sum scores of beliefs were strongly negatively correlated with this measure, *r*_GMfood,W1_ = − 0.44, *P* < 0.001, *r*_GMfood,W2_ = − 0.45, *P* < 0.001, *r*_GMfood,W3_ = − 0.45, *P* < 0.001, *r*_vaccines,W1_ = − 0.45, *P* < 0.001, *r*_vaccines,W2_ = − 0.46, *P* < 0.001, *r*_vaccines,W3_ = − 0.47, *P* < 0.001. Participants who were more negative toward GM food or childhood vaccinations reported a more negative affect on our measurement of felt dissonance.

To further test the validity of the model, we compared the estimated and self-reported centrality of moral and beliefs. This allowed us to test whether the estimated network structure was in line with the subjective perception by the participants. For this analysis, we made use of additional questions in our data. Participants rated how important their different beliefs are to their belief about the safety of GM food or childhood vaccines. Participants also rated to what extent they believed that GM food or childhood vaccines were safe. For this analysis, we reestimated the belief network including the safety beliefs. The results of this analysis are shown in fig. S4. We found that the estimated measures of centrality in relation to the safety of GM food and childhood vaccines were positively correlated with the self-reported importance of moral and social beliefs for safety.

### Statistical analysis

#### Correlation between network dissonance and average belief change

To test whether belief network dissonance predicts average belief change, we first correlated belief network dissonance and absolute belief change separately for each intervention and for each topic (these correlations and their associated significance levels can be found in table S4). We then transformed these Pearson’s correlation into Fisher’s *z* scores and entered the scores into a random effect meta-analyses. Last, we retransformed the Fisher’s *z* scores back to correlation coefficients for ease of interpretation.

#### Correlation between belief-specific dissonance and belief-specific change

To test whether belief-specific dissonance predicts belief-specific change, we first correlated belief-specific dissonance and belief-specific change separately for each intervention and for each topic (these correlations and their associated significance levels can be found in table S5). We then transformed these Pearson’s correlations into Fisher’s *z* scores and entered the scores into a random effect meta-analysis. Last, we retransformed the Fisher’s *z* scores back to correlation coefficients for ease of interpretation.

#### Differences between network dissonance before and after the educational interventions

To test whether belief network dissonance decreases after the interventions, we first calculated the mean differences between network dissonances before and after each intervention separately for each topic and intervention (these mean differences and their associated significance levels can be found in table S6). We then transformed these scores into standardized mean change scores and entered the scores into a random effect meta-analysis. Last, we retransformed the standardized mean change scores back to raw differences for ease of interpretation.
