# Supplementary material for: Using a cognitive network model of moral and social beliefs to explain belief change
Source: Sci Adv. 2022 Aug 19;8(33):eabm0137. doi: 10.1126/sciadv.abm0137 (PMC9390990; doi:10.1126/sciadv.abm0137)
Supplement: Supplementary file 1 — Tables S1 to S6 Figs. S1 to S5 [file sciadv.abm0137_sm.pdf]

Science Advances
AAAS

Supplementary Materials for

Using a cognitive network model of moral and social beliefs to explain belief change

Jonas Dalege and Tamara van der Does

Corresponding author: Jonas Dalege, j.dalege@santafe.edu

Sci. Adv. **8**, eabm0137 (2022)
DOI: 10.1126/sciadv.abm0137

This PDF file includes:

Tables S1 to S6
Figs. S1 to S5

Supplementary Materials

Supplementary Table S1: Questions about moral and social beliefs related to scientific issues (GM food or childhood vaccines).

**Moral**	**Social**
Instructions: For each of the following considerations, select a number between 1 and 7 that most closely represents your opinion.	Instructions: As far as you know, what is the percentage of people within the following groups who believe *scientific issue* is safe to eat for healthy children? Please give your best estimate.
scientific issue is not part of our tradition, or that it is part of our tradition?	% of medical doctors believe *scientific issue* is safe.
Producing *scientific issue* is harmful or beneficial to the environment?	% of representatives of governmental agencies believe *scientific issue* is safe.
scientific issue negatively or positively affects your family?	% of my family and close friends believe *scientific issue* is safe.
scientific issue is not approved by all the appropriate agencies, or that it is approved by all the appropriate agencies?	% of the US general public believe *scientific issue* is safe.
Some important information about *scientific issue* is not shared with the public, or that all important information is shared?	% of my online community believe *scientific issue* is safe.
Big biotechnology/pharmaceutical companies benefit more from *scientific issue* than farmers/patients, or that both big biotechnology/pharmaceutical companies and farmers/patients benefit from *scientific issue*?	% of online experts and influencers I follow believe *scientific issue* is safe.
Large-scale farmers/medical doctors benefit more from *scientific issue* than small-scale farmers/patients, or that both large-scale/medical doctors and small-scale farmers/patients benefit from *scientific issue*?	% of US scientists believe *scientific issue* is safe.
scientific issue is unnatural or natural?	% of journalists at your favorite news outlet believe *scientific issue* is safe.
You are forced to eat/use *scientific issue* without your consent, or that you are free to choose whether to eat/use *scientific issue*?	

God disapproves or approves of *scientific issue*?	
scientific issue is harmful or beneficial to children?	
scientific issue negatively or positively affects our country?	

Supplementary Table S2: Experimental groups and educational interventions.

**GM experiment**	**Vaccines experiment**
Nothing N=102	Nothing N=89
Simple message N=90<br>There is no evidence that GM food currently on the market is harmful when consumed by people or farm animals. This information comes from a summary of recent studies compiled by the National Academies of Science, Engineering, and Medicine.<br>These studies investigated evidence about the effects of GM food when consumed by people or farm animals. This summary comes in the form of a written report.	Simple message N=84<br>There is no evidence that currently recommended childhood vaccines cause long-term harm to healthy children. This information comes from a summary of recent studies compiled by the National Academies of Science, Engineering, and Medicine.<br>These studies investigated evidence about the effects of childhood vaccines on children's health. This summary comes in the form of a written report.
Scientists N=86<br>There is no evidence that GM food currently on the market is harmful when consumed by people or farm animals. This information comes from a summary of recent studies compiled by the National Academies of Science, Engineering, and Medicine.<br>A survey of the largest scientific society in the country revealed that 88 % of US scientists believe that it is safe to eat GM food.	Scientists N=82<br>There is no evidence that currently recommended childhood vaccines cause long-term harm to healthy children. This information comes from a summary of recent studies compiled by the National Academies of Science, Engineering, and Medicine.<br>A survey of the largest scientific society in the country revealed that 86 % of US scientists believe that all children should be required to be vaccinated.
Tradition (authority) N=90<br>There is no evidence that GM food currently on the market is harmful when consumed by people or farm animals. This information comes from a summary of recent studies compiled by the National Academies of Science, Engineering, and Medicine.<br>The production of GM crops imitates traditional farming techniques. These studies explain how all food crops have been modified from their wild relatives by farmers for thousands of years.	Tradition (authority) NA
Individual farmers (fairness) N=90	Corporations and individuals (fairness) N=88

There is no evidence that GM food currently on the market is harmful when consumed by people or farm animals. This information comes from a summary of recent studies compiled by the National Academies of Science, Engineering, and Medicine.<br>Both big biotechnology companies and farmers benefit from GM crops. These studies show how adopting GM crops increases yield and reduces costs for both large-scale and small-scale farmers.	There is no evidence that currently recommended childhood vaccines cause long-term harm to healthy children. This information comes from a summary of recent studies compiled by the National Academies of Science, Engineering, and Medicine.<br>Big pharmaceutical companies do not benefit from vaccines at the expense of patients. These studies explain how companies would profit more from people getting sick than from selling vaccines.
Information (freedom) N=91<br>There is no evidence that GM food currently on the market is harmful when consumed by people or farm animals. This information comes from a summary of recent studies compiled by the National Academies of Science, Engineering, and Medicine.<br>This information is shared with the public. This organization maintains a website which includes a list of reports on research conducted about GM food (https://www.nap.edu/topic/298/agriculture/crop-and-plant-production).	Information (freedom) N=87<br>There is no evidence that currently recommended childhood vaccines cause long-term harm to healthy children. This information comes from a summary of recent studies compiled by the National Academies of Science, Engineering, and Medicine.<br>This information is shared with the public. This organization maintains a website which includes a list of reports on research conducted about childhood vaccines. (https://www.nap.edu/collection/55/vaccines)

Supplementary Table S3: Fit measures of network model specifications.

Model	DF (GM food)	BIC (GM food)	DF (Childhood vaccines)	BIC (Childhood vaccines)
All parameters free (dense)	0	11162.88	0	4236.69
All parameters free (sparse)	557	7546.51	552	768.09
Equal networks (dense)	570	7557.98	570	863.80
Equal networks (sparse)	679	6862.13	680	164.47
Equal networks and thresholds (dense)	630	7096.31	630	406.94
Equal networks and thresholds (sparse)	739	6400.47	740	-282.53
All parameters equal (dense)	690	8275.81	690	1507.71
All parameters equal (sparse)	798	7567.16	801	167.19

DF: Degrees of Freedom due to constraints of the model specifications.
BIC: Bayesian Information Criterion.

Supplementary Table S4: Correlations between network dissonance and absolute belief change

Intervention	Correlation within Wave 2	Correlation Wave 2b and Wave 3
Gm food		
Control	$r = .30, t(87) = 2.91, p = .005$	$r = .20, t(87) = 1.95, p = .055$
Information	$r = .35, t(91) = 3.57, p < .001$	$r = .12, t(91) = 1.19, p = .237$
Farmers	$r = .09t(90) = 0.82, p = .414$	$r = .12, t(90) = 1.10, p = .274$
Scientists	$r = -.12, t(85) = -1.10, p = .275$	$r = .02, t(85) = 0.18, p = 860$
Tradition	$r = .16, t(92) = 1.57, p = .119$	$r = .07, t(92) = 2.91, p = .515$
Simple	$r = .28, t(92) = 2.75, p = .007$	$r = .23, t(92) = 2.24, p = .028$
Childhood vaccines		
Control	$r = .14, t(80) = 1.24, p = .217$	$r = .09, t(80) = 0.83, p = .409$
Information	$r = .07, t(85) = 0.64, p = 0.521$	$r = .04, t(85) = 0.40, p = .691$
Scientists	$r = 0.56, t(83) = 6.16, p < .001$	$r = .12, t(83) = 1.13, p = .263$
Simple	$r = .45, t(83) = 4.53, p < .001$	$r = .01, t(83) = 0.07, p = .947$
Big corporations	$r = .20, t(89) = 1.92, p = .058$	$r = .32, t(89) = 3.28, p = .001$

Supplementary Table S5: Correlations between belief-specific dissonance and absolute belief-specific change

Intervention	Correlation within Wave 2	Correlation Wave 2b and Wave 3
Gm food		
Control	$r = .22, t(1778) = 9.44, p < .001$	$r = .27, t(1778) = 11.81, p < .001$
Information	$r = .15, t(1858) = 6.33, p < .001$	$r = .14, t(1858) = 5.92, p < .001$
Farmers	$r = .28t(1838) = 12.73, p < .001$	$r = .16, t(1838) = 6.83, p < .001$
Scientists	$r = .17, t(1738) = 7.10, p < .001$	$r = .20, t(1738) = 8.50, p < .001$
Tradition	$r = .23, t(1878) = 10.32, p < .001$	$r = .15, t(1878) = 6.71, p < .001$
Simple	$r = .23, t(1878) = 10.27, p < .001$	$r = .16, t(1878) = 7.03, p < .001$
Childhood vaccines		
Control	$r = .26, t(1638) = 10.86, p < .001$	$r = .23, t(1638) = 9.39, p < .001$
Information	$r = .22, t(1738) = 9.31, p < .001$	$r = .16, t(1738) = 6.55, p < .001$
Scientists	$r = 0.29, t(1698) = 12.66, p < .001$	$r = .24, t(1698) = 10.14, p < .001$
Simple	$r = .26, t(1698) = 10.89, p < .001$	$r = .19, t(1698) = 8.07, p < .001$
Big corporations	$r = .18, t(1818) = 7.98, p < .001$	$r = .21, t(1818) = 8.98, p < .001$

Supplementary Table S6: Mean differences between network dissonance before and after interventions

Intervention	Difference Wave 2a and Wave 2b	Difference Wave 2b and Wave 3
Gm food		
Control	$MD = 0.16, t(88) = 3.10, p = .003$	$MD = 0.03, t(88) = 0.44, p = .663$
Information	$MD = 0.04, t(92) = 0.75, p = .455$	$MD = -0.02, t(92) = -0.15, p = .882$
Farmers	$MD = 0.23, t(91) = 2.47, p = .015$	$MD = 0.03, t(91) = 0.30, p = .763$
Scientists	$MD = 0.01, t(86) = 0.15, p = .884$	$MD = 0.18, t(86) = 1.78, p = .079$
Tradition	$MD = 0.29, t(93) = 3.67, p < .001$	$MD = -0.07, t(93) = -0.69, p = .492$
Simple	$MD = 0.19, t(93) = 2.51, p = .014$	$MD = 0.10, t(93) = 1.26, p = .211$
Childhood vaccines		
Control	$MD = 0.29, t(81) = 4.61, p < .001$	$MD = -0.06, t(81) = -0.71, p = .480$
Information	$MD = 0.23, t(86) = 3.90, p < .001$	$MD = -.03, t(86) = -0.41, p = .683$
Scientists	$MD = 0.34, t(84) = 3.84, p < .001$	$MD = -0.04, t(84) = -0.43, p = .669$
Simple	$MD = 0.24, t(84) = 3.42, p < .001$	$MD = -0.02, t(84) = -0.27, p = .786$
Big corporations	$MD = 0.25, t(90) = 4.60, p < .001$	$MD = -0.15, t(90) = -1.55, p = .126$

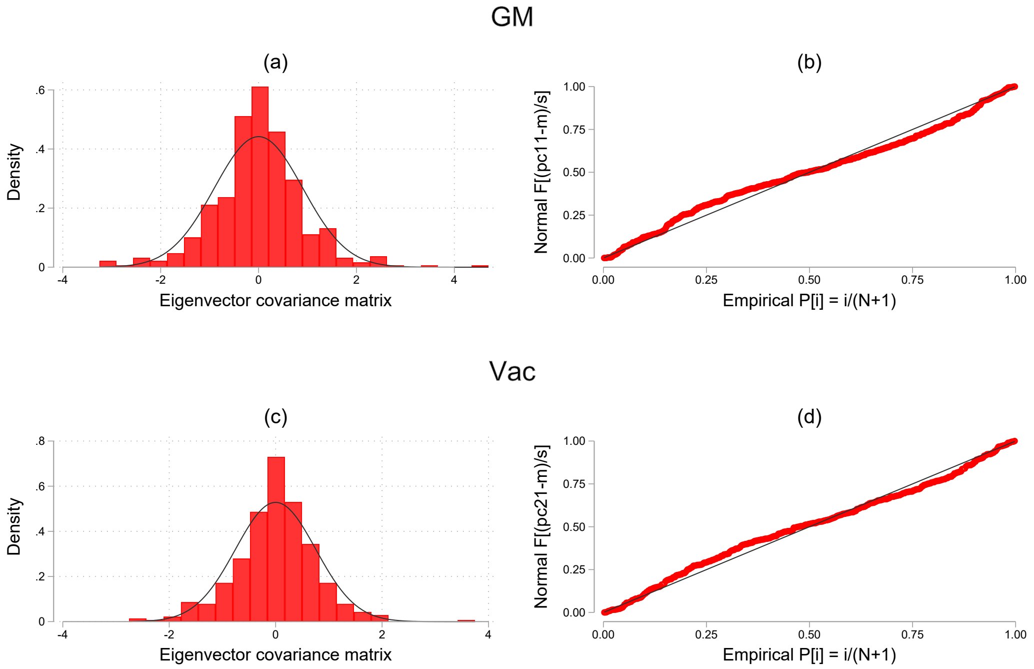

Supplementary Figure S1: Distribution of the covariance matrix eigenvector (principal component of moral and social beliefs across time points) compared to the normal distribution (a and c) and comparison between the eigenvector probability plot with the normal probability plot (b and d), for GM (a and b) and vaccines (c and d). In red are empirical data, N=979.

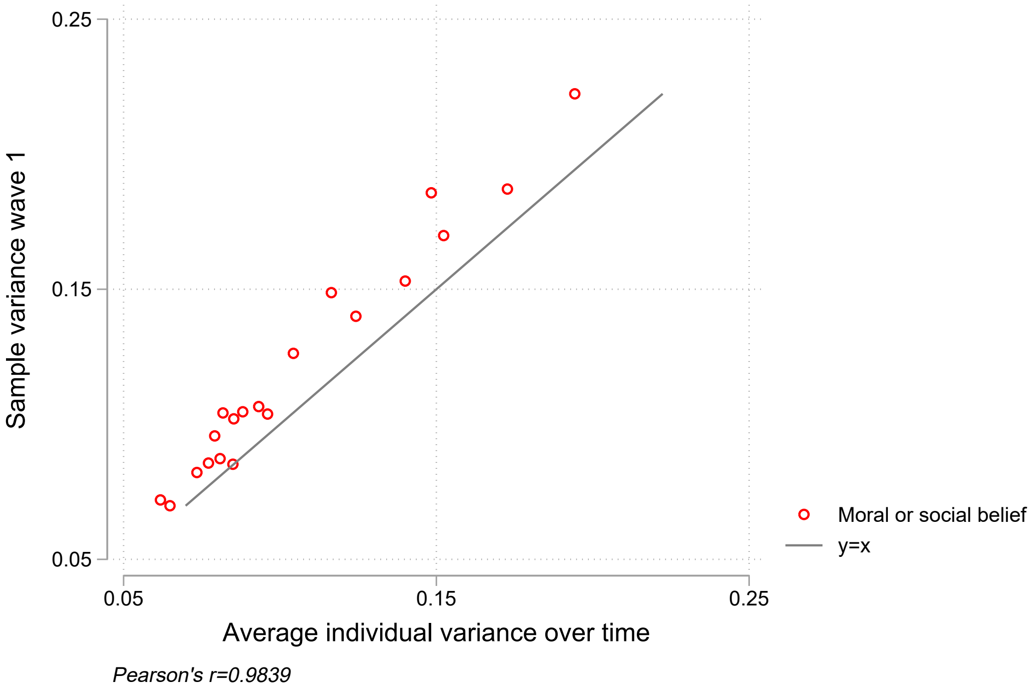

Supplementary Figure S2: Relationship between individual-level (over time) and population-level (across individuals at one time point) variances for each moral and social belief. Each dot represents one moral or social belief.

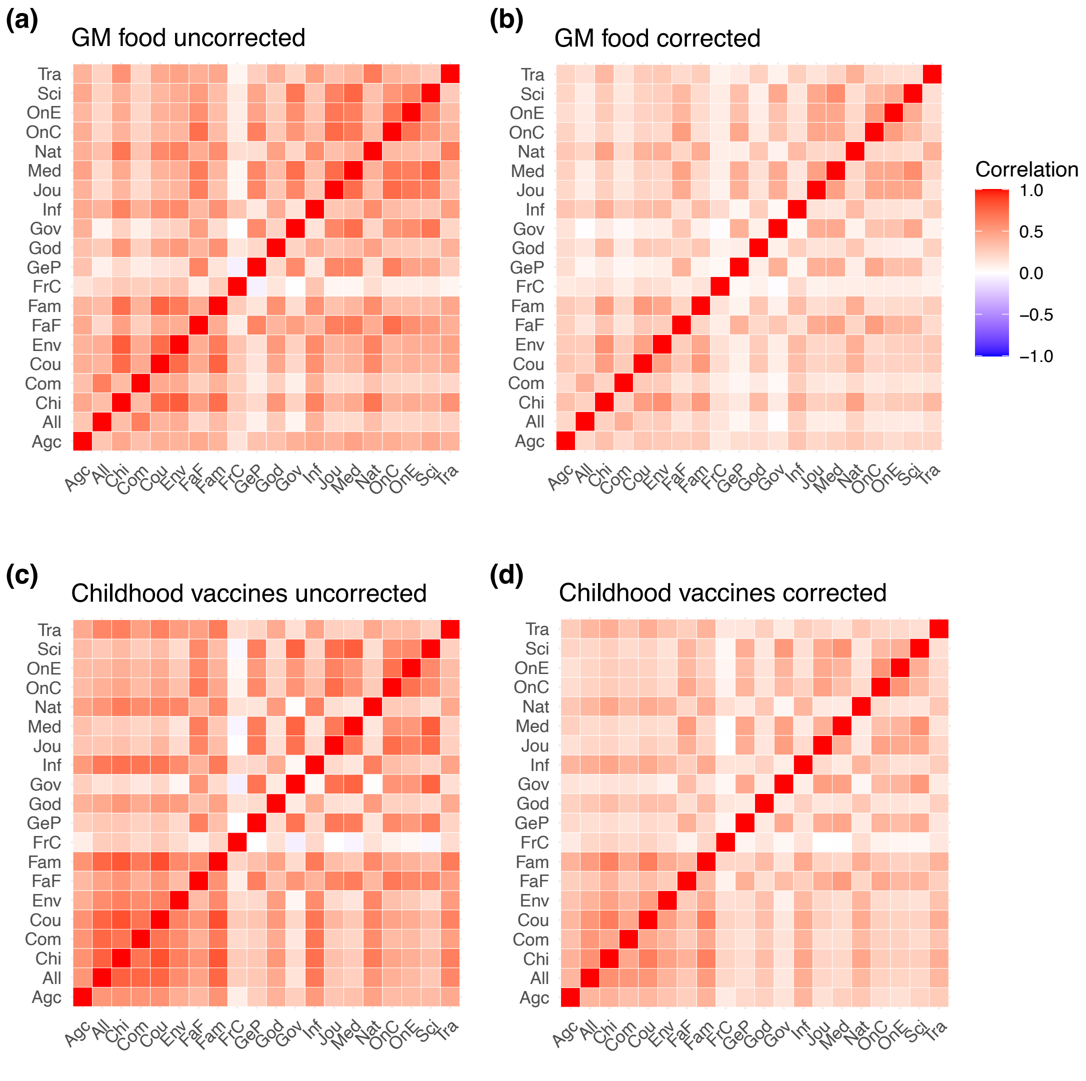

Supplementary Figure S3: Heatmaps of correlations uncorrected for the multilevel nature of the data (a, c) and corrected for the multilevel nature of the data, with time points nested within individuals (b, d) for GM food (a, b) and childhood vaccines (c, d). A higher density of redness represents stronger correlation coefficients.

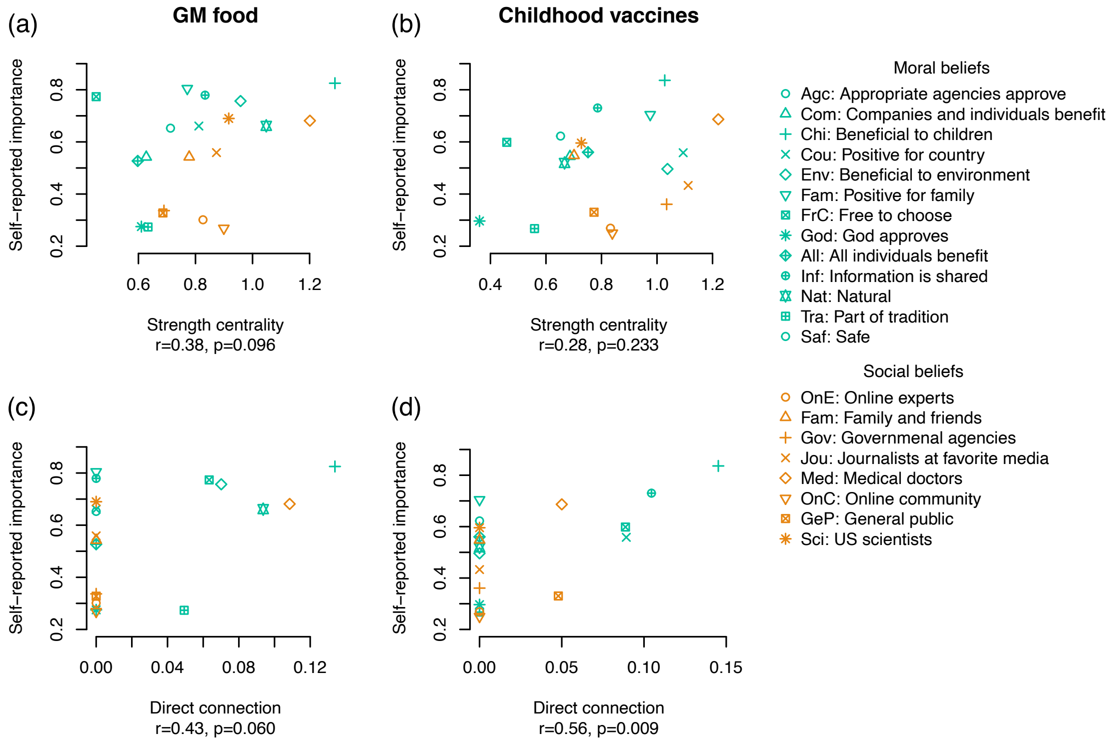

Supplementary Figure S4: Relationships between the centrality of beliefs in networks and the self-reported importance of beliefs for the safety belief. (a) shows the relationship between strength centrality and importance for GM food. (b) shows this relationship for childhood vaccines. (c) shows the relationship between the magnitude of the direct connection to the safety belief and importance for GM food. (d) shows this relationship for childhood vaccines.

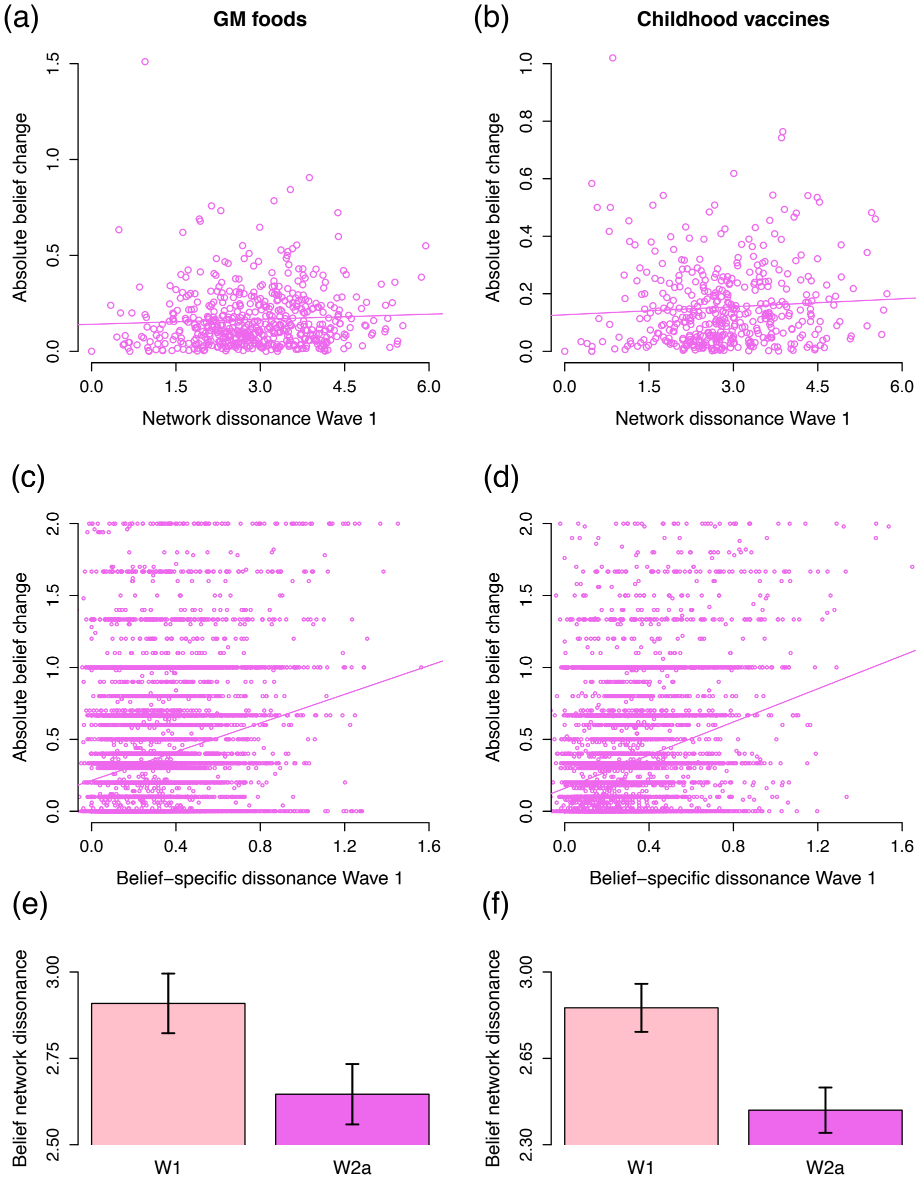

Supplementary Figure S5: Network dissonance and belief change between Wave 1 and Wave 2a. (a) and (b) show the relationship between network dissonance and absolute belief change for GM food ($r = .06, p = .177$), and childhood vaccines ($r = .07, p = .161$), respectively, where each dot represents a participant. (c) and (d) show the relationship of belief-specific dissonance and absolute belief-specific change for GM food ($r = .22, p < .001$), and childhood vaccines ($r = .27, p < .001$), respectively, where each dot represents a single belief of a participant. (e) and (f) show the belief network dissonance at Wave 1 and Wave 2 for GM food (difference: $t(548) = 6.02, p < .001$), and childhood vaccines (difference: $t(429) = 8.59, p < .001$), respectively. The error bars in (e) and (f) indicate 95% confidence intervals, N=979.
